# The United States Farriery, and Zoological History of Horses, Cattle, Sheep and Hogs; with Engravings; Connected with the Therapeutical Illustrations of Medicine

**Published:** 1848-04

**Authors:** 


Art. IX.—*The United States Farriery, and Zoological History of Horses, Cattle, Sheep and Hogs.* Connected with the Therapeutical illustrations of Medicine. Compiled from the most approved Authors, by Buell Eastman, M.D., Author of a New Medical Practice and Treatise on Human Diseases. Cincinnati: C. Cropper and Son: 1848. 184 pp. 16mo.

The subject of this little volume, we hesitate not to say, is one of dignity, and ought to find favor with every humane and enlightened mind. It is as clearly our duty as it is our interest, to study the diseases of the animals associated with us in our destiny, and upon which the comfort and progress of human society depend. Our readers have been informed, by the letter of Dr. Brooks from Paris, published in a former Number, how great is the attention paid in France to Veterinary surgery, and there are satisfactory indications that the subject is growing upon the attention of our brethren in America. The volume before us is not one of the highest class, but it contains valuable information on many important points, and being cheap, is likely to circulate extensively and do much good. It embraces in the first part, a full catalogue of the diseases of the horse, with their remedies. The second part is devoted to cattle and their diseases; the third embraces sheep, and the fourth hogs. Besides the matter strictly professional, the author has given in his work much other useful information, and has interspersed it with pertinent remarks on our duty to animals.

